# Low Pathogenic Avian Influenza Isolates from Wild Birds Replicate and Transmit via Contact in Ferrets without Prior Adaptation

**DOI:** 10.1371/journal.pone.0038067

**Published:** 2012-06-01

**Authors:** Elizabeth A. Driskell, Jennifer A. Pickens, Jennifer Humberd-Smith, James T. Gordy, Konrad C. Bradley, David A. Steinhauer, Roy D. Berghaus, David E. Stallknecht, Elizabeth W. Howerth, Stephen Mark Tompkins

**Affiliations:** 1 Department of Pathology, University of Georgia, Athens, Georgia, United States of America; 2 Department of Infectious Diseases, University of Georgia, Athens, Georgia, United States of America; 3 Department of Microbiology and Immunology, Emory University School of Medicine, Atlanta, Georgia, United States of America; 4 Department of Population Health, University of Georgia, Athens, Georgia, United States of America; Virginia Polytechnic Institute and State University, United States of America

## Abstract

Direct transmission of avian influenza viruses to mammals has become an increasingly investigated topic during the past decade; however, isolates that have been primarily investigated are typically ones originating from human or poultry outbreaks. Currently there is minimal comparative information on the behavior of the innumerable viruses that exist in the natural wild bird host. We have previously demonstrated the capacity of numerous North American avian influenza viruses isolated from wild birds to infect and induce lesions in the respiratory tract of mice. In this study, two isolates from shorebirds that were previously examined in mice (H1N9 and H6N1 subtypes) are further examined through experimental inoculations in the ferret with analysis of viral shedding, histopathology, and antigen localization via immunohistochemistry to elucidate pathogenicity and transmission of these viruses. Using sequence analysis and glycan binding analysis, we show that these avian viruses have the typical avian influenza binding pattern, with affinity for cell glycoproteins/glycolipids having terminal sialic acid (SA) residues with α 2,3 linkage [Neu5Ac(α2,3)Gal]. Despite the lack of α2,6 linked SA binding, these AIVs productively infected both the upper and lower respiratory tract of ferrets, resulting in nasal viral shedding and pulmonary lesions with minimal morbidity. Moreover, we show that one of the viruses is able to transmit to ferrets via direct contact, despite its binding affinity for α 2,3 linked SA residues. These results demonstrate that avian influenza viruses, which are endemic in aquatic birds, can potentially infect humans and other mammals without adaptation. Finally this work highlights the need for additional study of the wild bird subset of influenza viruses in regard to surveillance, transmission, and potential for reassortment, as they have zoonotic potential.

## Introduction

The host and virulence range for avian influenza viruses (AIV) continues to surprise, with numerous cases of direct transmission from birds to mammals that result in a range of disease including pneumonia, conjunctivitis, and occasionally systemic disease [1]–[3]. Although transmission of AIV to humans resulting in disease has been limited to poultry adapted viruses, there is evidence of both direct transmission of AIV to other mammalian species [3]–[5] and experimental evidence that numerous AIV hemagglutinin (HA) subtypes can infect mammals [5]–[10]. However, there remains a great void of knowledge regarding the capacity of AIV to infect mammals, especially related to AIVs from the wild bird reservoir. Human AIV infections have been limited to the H5, H7, and H9 subtypes [2], [11]–[14] and these viruses are of concern because they have a pandemic potential if they become highly transmissible in the human population. Recent studies have demonstrated the high compatibility of avian and human influenza reassortants *in vitro* and *in vivo* and generation of viable reassortants *in vivo* in ferrets, further raising the concern of the natural generation of a pandemic strain [15]–[18]. Examining the capacity of a spectrum of wild bird AIVs to infect mammals is necessary to complete our understanding of AIV host range restrictions and to better define potential risks of mammalian infection and viral reassortment.

We have previously screened wild bird AIVs in a mouse model and demonstrated their varying capacity to replicate in the lung of mice, with some isolates exhibiting robust pulmonary replication regardless of HA subtype and causing mild clinical disease [19]. Ferrets are a better model for influenza infection and transmission in humans as they are naturally susceptible to the virus and have a similar distribution of sialic acid glycans in the respiratory tract; they have also been used for numerous studies of AIV that have resulted in human disease [20]–[22]. In this study, two wild bird AIVs (H1N9 and H6N1 subtypes) that exhibited robust pulmonary replication in mice were further studied in a ferret model to better assess pathogenesis and transmission capacity in mammals.

Viral contributors to host range restriction and virulence of AIVs in mammals have been demonstrated to be a multifactorial. The interaction between the major viral glycoprotein, the hemagglutinin (HA) and the host cell sialic acid receptors is considered critical for establishing an influenza infection, and species specific binding restrictions have been identified. Influenza viruses of avian origin preferentially bind terminal sialic acids with a α2,3 linkage located in cells in the gastrointestinal tract of birds and on the ciliated cells and type II pneumocyte in the human respiratory tract [23]–[28]. Conversely, human influenza viruses exhibit preferential binding to terminal sialic acids with a α2,6 SA linkage located most prominently on non-ciliated cells of the human upper respiratory tract (nasopharynx and trachea) [23], [27], [29]–[32]. It is thought that the receptor specificity of influenza viruses is a large component of host restriction; where in some AIV cases (H5, H7, and H9 subtypes), the viruses are able to infect and cause disease in humans yet exhibit poor human to human transmission [14], [33]–[35].

The amino acid residues contributing to α2,3 versus α2,6 SA binding specificity have been described for some viruses and mutation analysis has shown some of these residues to be directly involved with altering viral receptor specificity. In human H3 strains, amino acids Leu226 and Ser228 (H3 numbering) results in α2,6 SA binding, where avian strains that preferentially bind α2,3 SA receptors exhibit a Gln226 and Gly228 amino acid sequence [26], [27], [32], [36]. Amino acid residues 138, 190, 194, and 225 (H3 numbering) have also been shown to be differentially conserved in avian and human influenza viruses [32].

Here we examine the potential for infection and transmission of an H1N9 (A/Ruddy Turnstone/DE/1171/02abbreviated H1N9) and an H6N1 (A/Ruddy Turnstone/DE/892/02abbreviated H6N1) AIV. Using sequence, *in vitro* binding, and glycan microarray analysis we determined the receptor specificity of these viruses. Using the ferret model, the most representative animal model of human influenza virus infection, we performed *in vivo* assessment of the potential for infection, replication, and transmission of these viruses in mammals. Despite a dominant α2,3 (avian) binding specificity we demonstrate that both of these viruses replicate in both the upper and lower respiratory tract of ferrets, inducing pulmonary lesions, but resulting in little morbidity. Moreover, we demonstrate that one of these viruses (H1N9) is able to transmit via direct contact, despite its dominant avian α2,3 SA binding preference. These findings support the recent findings of others [37] and taken together demonstrate that AIV circulating in wild bird populations can transmit to mammals, albeit not always causing clinical disease, and that transmissibility of AIV to mammals is not restricted to specific subtypes (i.e. H5N1 or H7). Further investigation of these circulating AIVs is warranted to better understand their zoonotic potential.

## Results

### Wild bird influenza viruses replicated in ferrets but exhibited low virulence

Infection in H1N9 and H6N1 inoculated ferrets and in H1N9 contact ferrets was demonstrated with presence of virus in nasal washes and seroconversion despite minimal clinical signs (Table 1). Three ferrets infected with H6N1 had transient, mild weight loss that was most prominent day 1 pi (Table 1), but two of these ferrets took an additional two to six days to return to pre-infection weight. In contrast, six out of seven ferrets infected with H1N9 had mild weight loss most prominent at day 1 post inoculation (pi) (Table 1) in which it took up to four days to return to pre-infection weight. The average temperature of H1N9 and H6N1 inoculated ferrets was most elevated on day 1 pi (Table 1). Direct contact H1N9 ferrets had elevated temperatures that did correlate with shedding of virus in nasal washes, while direct contact H6N1 ferrets, which did not become infected, had rare, inconsistent temperature elevations. Sneezing was not observed in any group of ferrets and all ferrets remained bright and alert through the duration of the study. There were no statistically significant differences in the total or differential leukocyte parameters for group by day interactions. However, mean lymphocytes (mean+/−standard error) decreased in both H6N1 (3.50+/−0.06 pre inoculation to 3.26+/−0.06 day 1 pi) and H1N9 (3.76+/−0.06 pre inoculation to 3.61+/−0.09 day 1 pi) inoculated groups compared to the allantoic inoculated group (3.46+/−0.05 pre inoculation to 3.45+/−0.06 day 1 pi) for day 1 pi, and then increased for the H1N9 (4.06+/−0.16 day 7 pi) inoculated group compared to the allantoic inoculated group (3.67+/−0.11 day 7 pi) and remained elevated through day 18 pi (Table S1).

**Table 1:** Morbidity, seroconversion, and respiratory viral replication of ferrets inoculated with wild bird avian influenza viruses H1N9 and H6N1.

Inoculated animals							
	Clinical parameters			Virus shedding		Seroconversion	
Virus	Weight loss (max %, average %)	Range temperature increase^a^	Sneezing	Virus detection in nasal wash (peak log_10_ TCID_50_/mL)^b^	Average TCID50/g virus in lung (day p.i.)^c^	Number with seroconversion (HI titers)	Number with seroconversion (MN titers)
H6N1	3/7 (6.3, 5.8)	0.1–1.6	0/3	7/7 (5.2)	Not detected (3,7)	3/3 (1∶160, 1∶160, 1∶160)	3/3 (1∶2560, 1∶1280, 1∶640)
H1N9	6/7 (12.8, 4.5)	0.1–1.7	0/3	7/7 (5.8)	5.1 (2); Not detected (3,7)	3/3 (1∶40, 1∶40, 1∶40)	3/3 (1∶320, 1∶640, 1∶320)
Allantoic fluid	0/3	0.1–2.4^d^	0/3	0/3	ND^e^	0/3^f^	ND

Direct contact animals							
H6N1	1/3 (2.7, <0)	0.4–1.4	0/3	0/3	ND	0/3	0/3
H1N9	2/3 (6.0, <0)	0.1–1.5	0/3	3/3 (6.0)	ND	3/3 (1∶20, 1∶40, 1∶80)	3/3 (1∶320, 1∶640, 1∶640)

a Temperature is in degrees Celsius.

b Limit of detection for nasal wash 1.5 log_10_ TCID_50_/mL.

c Limit of detection for lung day 7 pi both viruses and day 3 pi for H6N1 is 1.3 TCID_50_/g; for days 2 and 3 pi for H1N9 is 1.0 TCID_50_/g.

d One control ferret potentially had an unrelated infection, but remained influenza seronegative.

e Not done.

f Tested against both H6N1 and H1N9.

### Both H1N9 and H6N1 wild bird influenza viruses replicated in the upper respiratory tract of ferrets

**Figure 1:**
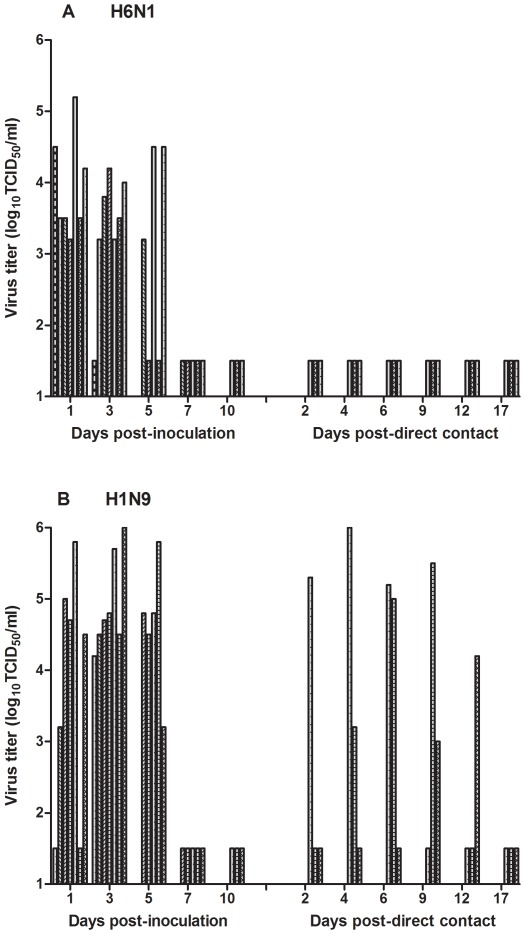
Nasal shedding and direct contact transmission of wild bird influenza viruses in ferrets. Seven ferrets were intranasally inoculated with 5×10^5^ PFUs of either H6N1 (A) or H1N9 (B) and nasal washes were collected and titered on MDCK cells (days post-inoculation portion of graph). Three naïve ferrets were paired with three of the inoculated ferrets 24 hours post inoculation for each virus group (days post-direct contact portion); nasal washes were collected titered on MDCK cells. Both H6N1 and H1N9 demonstrated replication in the upper respiratory tract of the ferrets, however, viral shedding was consistently greater in magnitude and duration for H1N9. H1N9 demonstrated direct contact transmission, but H6N1 did not transmit to direct contact ferrets.

**Figure 2:**
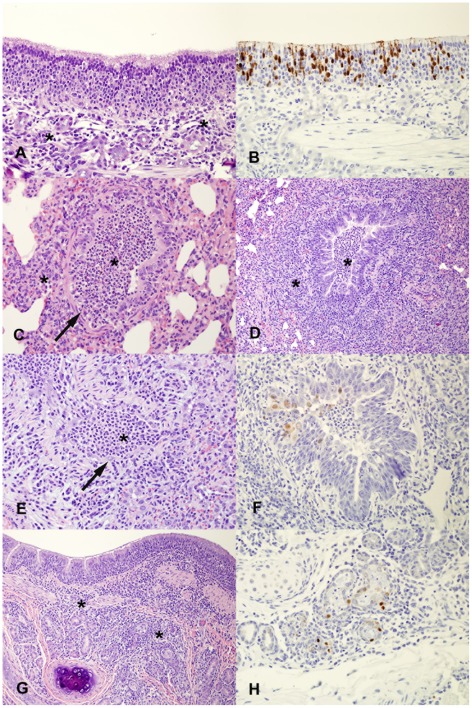
Histopathologic lesions and influenza antigen localization in ferrets inoculated with wild bird influenza viruses. A) Nasal turbinates of ferrets inoculated with H6N1 or H1N9 demonstrated moderate submucosal inflammation (asterisk) (H1N9, d3pi). B) There is widespread strong intranuclear and some intracytoplasmic positive immunoreactivity for the nucleoprotein of influenza A on immunohistochemistry in ferrets inoculated with H6N1 or H1N9 on day 3 pi (H1N9, d3pi). C) Epithelial damage in the lung was early for ferrets inoculated with H1N9, with necrosis in the bronchioles (arrow) and inflammation (asterisks) within and around bronchioles on day 2 pi. D and E) There was evidence of early repair with regeneration of bronchiolar epithelium (arrow) and persistence of inflammation (asterisks) on day 3 pi for ferrets inoculated with H6N1 and H1N9 (H1N9, d3pi). The arrow highlights the stretched and plump bronchiolar epithelial cells, indicating regeneration. F) Presence of influenza antigen was confirmed in ferrets inoculated with H6N1 and H1N9 by strong positive intranuclear staining of bronchiolar epithelial cells with immunohistochemistry on day 3 pi (H6N1, d3pi). G) Inflammation around larger airways in the lung was also present in ferrets inoculated with H6N1 and H1N9, with prominent periglandular bronchial inflammation (asterisk) (H6N1, d7pi). H) There was strong positive intranuclear staining for the nucleoprotein of influenza in the peribronchial glandular epithelial cells on immunohistochemistry on day 3 pi in ferrets inoculated with H6N1 and H1N9 (H1N9, d3pi).

Both H1N9 and H6N1 demonstrated replication in the upper respiratory tract of ferrets, evident by nasal washes and immunohistochemistry (IHC). Although both H1N9 and H6N1 replicated in the nasal cavity, H1N9 replication was more robust, reaching consistently higher titers than H6N1 for days 3 and 5 pi with shedding occurring consistently longer (Figure 1A, B and Table 1). Lesions of influenza infection were present in the nasal turbinates of ferrets infected with H1N9 and H6N1 (Figure 2A), however, only a single ferret in the H6N1 group (day 3 pi) had lesions while nasal turbinates of all four ferrets infected with H1N9 had lesions (both days 3 and 7 pi). This correlates with the viral titers obtained from nasal washes of these ferrets, as viral shedding quickly declined over a short period of time in this group with some ferrets shedding virus only up to day 3 pi (Figure 1). Lesions in the nasal turbinates included infiltration of the submucosa with mild to moderate numbers of lymphocytes with fewer plasma cells and occasional submucosal edema on both days 3 and 7 pi, with an increased amount of inflammation on day 7 pi for H1N9 (Figure 2A). The presence of influenza viral antigen was confirmed by abundant strong intranuclear and frequent intracytoplasmic staining on IHC for the nucleoprotein (NP) of influenza A in the nasal turbinates epithelium (Figure 2B). Positive staining occurred on day 3 pi in the nasal turbinates of ferrets that had lesions and was not present by day 7 pi for both viruses.

### Both H1N9 and H6N1 wild bird influenza viruses replicated in the lower respiratory tract of ferrets

There was evidence of replication of both H1N9 and H6N1 in the lung of inoculated ferrets, with similar histopathologic progression of lesions for both viruses. Influenza was detected in the lung for both viruses for all ferrets day 3 pi using virus isolation in ECEs, but was not isolated for day 7 pi from the lung for either group of ferrets (Table 2). For both viruses, histopathologic change in the lung on day 3 pi was characterized by a small amount of mucus admixed with neutrophils and macrophages within the lumens of bronchi and large bronchioles with peribronchial inflammation, primarily lymphocytes, that surrounded and occasionally infiltrated peribronchial glands (Figure 2G). There was positive intranuclear immunoreactivity against the NP of influenza A in small numbers of bronchiolar cells in the lungs of ferrets infected with H1N9 and H6N1 on day 3 pi (Figure 2F). No influenza antigen was present via IHC in the lung of ferrets infected with either of the two viruses on day 7 pi, supporting that virus was cleared from the lung by that time point. Rare peribronchiolar glands contained cellular debris and necrosis of the glandular epithelium, with presence of influenza antigen in the epithelial cells demonstrated via IHC on day 3 pi (Figure 2H). Smaller bronchioles were frequently filled with neutrophils, macrophages, and cellular debris and lined by a mixture of ectatic and very plump epithelial cells, indicative of early repair after previous epithelial damage (Figure 2D, E). Smaller bronchioles still had intraluminal inflammatory exudate and epithelial regeneration on day 7 pi. Peribronchiolar alveoli of affected bronchioles were occasionally filled by macrophages, but notably, there were minimal alveolar changes. Some pulmonary vessels had small perivascular cuffs composed primarily of lymphocytes. On both day 3 and day 7 pi for both viruses, the caudal lung lobes were more affected in severity and extent than the cranial lung lobes. Importantly, only segmental areas of the lung were affected in ferrets inoculated with either virus, with some lung lobes in individual ferrets having no histopathologic lesions at all. There was evidence of viral replication in the trachea, with rare neutrophils present in the epithelium for both H1N9 and H6N1 and rare positive intranuclear epithelial immunoreactivity for the NP of influenza for one H1N9 inoculated ferret. Lung in the three inoculated ferrets from repeat transmission studies was examined microscopically at day 21 pi; these tissues had no significant lesions, indicating that complete resolution of pulmonary damage and inflammation had occurred by this time.

**Table 2:** Presence of influenza virus in ferret organs by virus isolation in ECEs for ferrets infected with wild bird avian influenza viruses H6N1 and H1N9.

Virus	Days post inoculation	Lung	Rectal swab	Intestine	Olfactory bulb	Liver	Spleen
**H6N1**	3	2/2	2/7	0/2	1/2	1/2	0/2
	5	ND^a^	1/5	ND	ND	ND	ND
	7	0/2	0/5	ND	ND	ND	ND
**H1N9**	3	2/2	1/7	0/2	0/2	2/2	1/2
	5	ND	1/5	ND	ND	ND	ND
	7	0/2	1/5	ND	ND	ND	ND

For each organ listed, the number of animals with a positive isolation of virus out of the number of animals tested is shown.

a Not done for this sample.

As there was evidence of pulmonary damage with regeneration, we suspected that viral replication in the lung had already peaked by day 3 pi. A titer could not be obtained for the lung via TCID_50_ assay for day 3 pi for either virus (limit of detection = 1.3 or 1.0 log_10_ TCID_50_/g) despite positive virus isolation. Therefore, two additional ferrets were inoculated with H1N9 and lung examined at day 2 pi. In the day 2 pi ferret lung, a virus titer was obtained using a 1∶3 dilution scheme (Table 1). Histopathologic changes in the lung on day 2 pi also confirmed suspicion of early viral replication, damage, and clearance, as the affected bronchiolar epithelium had necrosis and sloughing without indication of the regeneration that was observed on day 3 pi (Figure 2C). Positive intranuclear immunoreactivity against the NP of influenza A was also observed in small numbers of bronchiolar cells in the lungs for day 2 pi.

### Extra-respiratory detection of wild bird influenza viruses in ferrets

Few changes in other organs were observed on histopathology. There was mild to moderate gross enlargement of tracheobronchial lymph nodes with microscopic lesions of moderate follicular hyperplasia as a result of the antigenic stimulation in all ferrets (both H1N9 and H6N1, days 3 and 7 pi). Perivascular cuffs of lymphocytes were present in the olfactory nerves and olfactory bulbs of the brain for one of the two ferrets infected with H6N1 on day 7 pi and one of the two ferrets infected with H1N9 on day 7 pi, although no influenza antigen was detected via immunohistochemistry on day 3 pi in the olfactory bulb for any of the ferrets. However, these lesions in combination with positive virus isolation on olfactory bulb in one of the ferrets infected with H6N1 on day 3 pi, are supportive of probable direct extension of virus from the infected nasal epithelium, as has been shown in numerous experimental intranasal influenza inoculations (Table 2) [6], [21], [38]. Virus isolation was also performed on several other organs with sporadic positive results for rectal swabs, liver, and spleen, despite the absence of histopathologic lesions in these organs (Table 2).

### Wild bird H1N9 influenza virus transmitted via contact between ferrets

Interestingly, H1N9 exhibited contact transmission between ferrets consistently in all three ferret pairs, but H6N1 did not directly transmit (Figure 1). All three direct contact ferrets that became infected with H1N9 had similar peak viral titers (average peak of 5.2 log_10_ TCID_50_ for transmission ferrets and average peak of 5.4 log_10_ TCID_50_ for inoculated ferrets) with similar length of shedding time (5 days for both groups) in the nasal wash compared to inoculated ferrets, although the time point of transmission varied greatly between pairs. Transmission variability may be somewhat explained by the varied time point of peak virus in the inoculated ferrets, which matched the pattern of transmission to the paired direct contact ferret (e.g. the later the peak virus in the inoculated ferret, the later the paired contact ferret had indication of transmission). Again, in ferrets that were infected via direct contact transmission, ferret health including clinical signs and morbidity parameters (temperature/weight loss) were minimally affected (Table 1). Direct contact transmission of H1N9 was repeated with a second study, and subsequently confirmed by two out of three direct contact ferrets becoming infected (data not shown).

### Wild bird influenza viruses exhibited typical AIV receptor specificity

Effective transmission by the H1N9 virus raised questions regarding potential mechanisms for transmission. Segment four, encoding the HA gene was sequenced for each virus to examine receptor specificity as compared to HA sequences defined in the literature. Both H6N1 and H1N9 viruses contained glutamic acid (E) and glycine (G) at positions 190 and 225 of the HA, in contrast to human influenza strains, A/North Carolina/1/1918 and A/Pennsylvania/08/2008, that contains aspartic acid (D) at both 190 and 225 position (Table 3) that is associated with to α2,6 linked sialic acid receptor specificity [39], [40]. The glutamine (Q) at position 226 and glycine (G) at position 228 of the HA are characteristic of avian strains but have been also been identified in human strains and have been shown to influence α2,3 linked sialic acid receptor specificity. The H1N9 and H6N1 viruses contained the HA amino acid residues most commonly present in avian influenza strains that exhibit α2,3 linked sialic acid specificity.

**Table 3:** Comparison of critical amino acids involved in receptor specificity of influenza hemagglutinin.

				Hemagglutinin Amino Acids^a^				
Virus	Isolate ID	HA Subtype	Host	190	225	226	228	Accession No.
**A/Ruddy Turnstone/DE/1171/2002**	H1N9	H1	Avian	E	G	Q	G	CY116631
**A/Ruddy Turnstone/DE/892/2002**	H6N1	H6	Avian	E	G	Q	G	CY116633
**A/Duck/Alberta/35/1976**		H1	Avian	E	G	Q	G	AF091309
**A/South Carolina/1/1918**		H1	Human	D	D	Q	G	AF117241
**A/New Caledonia/20/1999**		H1	Human	N	D	Q	G	AB304818
**A/Pennsylvania/08/2008**		H1	Human	D	D	Q	G	FJ549047
**A/California/04/2009**		H1	Human	D	D	Q	G	FJ966082

a Hemagglutinin residues using H3 numbering.

To functionally assess the sialic acid receptor specificity of H1N9 and H6N1, we examined the erythrocyte binding of the avian influenza strains. Both viruses were able to agglutinate equine erythrocytes to a 512 HAU/ml titer (Table 4), while the human influenza strains (A/New Caledonia/20/1999 and A/California/04/2009) generated no detectable titer. Red blood cells (RBCs) from most species express both α2,3 and α2,6 linked sialic acids, but equine erythrocytes are unique in that they exhibit predominantly α2,3 linked SA receptors. Both H6N1 and H1N9 agglutinated guinea pig and turkey RBCs to similar levels as compared to a human influenza. Turkey erythrocytes contain a mixture of α2,3 and α2,6 linked sialic acid linked receptors and guinea pig RBCs express largely α2,6 linked sialic acids with lower levels of α2,3 linked sialic acid receptors [41]. While the mixed α2,3 and α2,6 linkages on these RBCs precludes determination of definitive α2,6 linked sialic acid binding, the lack of clear changes in binding to erythrocytes expressing predominantly α2,6 linked sialic acids suggests limited binding to these glycans.

**Table 4:** Agglutination of erythrocytes from different animal species by human and avian influenza viruses.

				Hemagglutination Titers^a^		
Virus	Isolate ID	HA Subtype	Host	Turkey	Equine	Guinea Pig
**A/Ruddy Turnstone/DE/1171/2002**	H1N9	H1	Avian	1024	512	512
**A/Ruddy Turnstone/DE/892/2002**	H6N1	H6	Avian	1024	512	256
**A/New Caledonia/20/1999**		H1	Human	1024	0	256
**A/California/04/2009**		H1	Human	64	0	16
**A/Pennsylvania/08/2008**		H1	Human	128	0	32

a Hemagglutination titers are provide as the reciprocal of the highest virus dilution generating agglutination.

**Figure 3:**
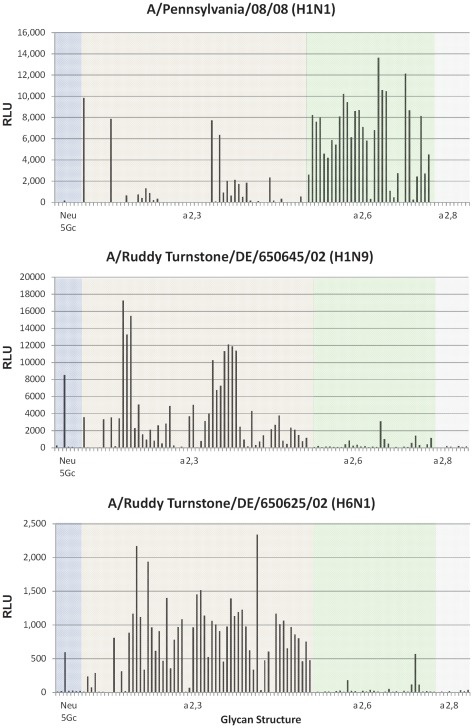
Glycan binding analysis of wild bird avian or human influenza viruses. Influenza viruses were propagated in Madin-Darby kidney cells, purified on a 25% sucrose cushion by ultracentrifugation, and labeled with Alexa488 before being applied to the microarray. The data was organized based on Neu5GC, α2,3 SA, α2,6 SA and α2,8 SA glycan structures and represented by different color schemes. Glycan microarray binding analysis was performed by Core H of the Consortium for Functional Glycomics. A) A/Ruddy Turnstone/DE/1171/02 (H1N9), B) A/Ruddy Turnstone/DE/892/02 (H6N1), C) A/Pennsylvania/08/2008 (H1N1).

To further define the receptor specificity of the viruses, glycan microarrays were utilized to determine the precise sialyl-oligosaccharide binding profile for H1N9 and H6N1 as compared to a previously defined seasonal H1N1 human influenza virus, A/Pennsylvania/08/2008 [40]. Purified and fluorescently labeled viruses were submitted to Core H of the Consortium for Functional Glycomics and binding was assessed against 511 glycans (Table S2). The binding motif for all three viruses shows that the H1N9 and H6N1 predominantly bind oligosaccharides that contain N-acetylneuraminic acid α2,3 moieties with little binding observed for the α2,6 and α2,8 linked sialic acid containing glycans (Fig. 3A,B). There was limited N-glyconeuraminic acid (NeuGc) recognition for the H1N9 and H6N1 with only the α2,3 NeuGc glycans demonstrated binding. The NeuGc moieties are the predominant sialic acid species on equine erythrocytes, where the H1N9 and H6N1 α2,3 NeuGc SA binding preference corroborates the horse erythrocyte agglutination data (Table 4) [42]. In contrast, the human control strain (A/Pennsylvania/08/2008) demonstrated the predicted α2,6 linked sialic acid receptor binding properties (Fig. 3C) with minor α2,3 linked sialic acid binding, which is common among human influenza viruses. This uncharacteristic α2,3 linked sialic acid receptor specificity is often the result of fucosylated and sulfated modifications to the oligosaccahrides enhancing suboptimal sialic acid receptor recognition, which, as previously shown, appears to be the case for the human control strain (A/Pennsylvania/08/2008) [40].

## Discussion

It has been demonstrated that some low pathogenic H6, H7, and H9 AIV subtypes have the ability to replicate in ferrets; however studies that have demonstrated this examined primarily poultry adapted isolates and have limited scope in examining the full pathogenesis of these infections [6], [7], [10], [43]. Van Hoven *et al* [43] demonstrated that a two H1N1 AIVs isolated a ducks and a mallard could infect ferrets and replicate to high titers in the upper respiratory tract of ferrets. We have previously shown that current circulating North American wild bird LPAIVs do have a capacity to infect and replicate in mammals using a mouse model of infection, although they caused little or no disease [19]. More recently, Nam *et al* [37] identified an H6N5 isolated from a fecal sample of an aquatic bird in Korea, A/AB/Kor/CN5/09 (H6N5), that infected mice, replicated to high titer and caused mortality without adaptation. Interestingly, this virus also disseminated to extrapulmonary tissues. From the previous work we selected two viruses of distinct subtype for further study in the ferret model, which most accurately emulated human infection and transmission of influenza virus [44]–[46]. We have expanded upon previous work by demonstrating that subtypes of “lesser concern” isolated from migratory shore birds (*i.e.* Ruddy Turnstone) can also directly infect and replicate in mammals, even with the potential for direct contact mammal to mammal transmission (Table 1, Figure 1). In the study by Nam *et al* the A/AB/Kor/CN5/09 (H6N5) virus also replicated to high titers in the upper respiratory tract of ferrets and caused middle disease. Similar to our findings with the H1N9 virus, the H6N5 virus showed limited transmission to contact naïve ferrets [37]. Interestingly, the PB1 segment of the H6N5 virus was similar to and possibly derived from the H5N1 highly pathogenic avian influenza viruses circulating in Asia. In contrast, comparison of the sequences from the AIVs described here demonstrates that the segments are all of LPAIV origin, unrelated to HPAI viruses (data not shown). The H6N5 virus was isolated from feces, and so the species of origin is unknown. It would be interesting to identify the potential reservoirs of this virus through live bird surveillance.

Our viruses were passaged in embryonated chicken eggs, which may lend to some adaptive mutations; however, we kept serial passages low to minimize alteration of the original viruses. Interestingly, these viruses replicated to relatively high titers in the upper respiratory tract of the ferret and induced lesions in both the upper and lower respiratory tracts, but minimal disease was observed clinically with complete resolution of pulmonary lesions (Table 1, Figures 1–2). Importantly, lesions and antigen localization in the lung indicate there was no alveolar replication of the virus. Alveolar localization of influenza with alveolar lesions has been associated with increased virulence [47]. Additionally, the results of virus titration and histopathology comparing days 2 and 3 pi is supportive that infection was rapid, minimal, and had rapid clearance in the lower respiratory tract. These observations may have played a role in the minimal induction of disease for these two viruses in the ferret. A study that examined a variety of AIVs of the H6 subtype not only demonstrated replication with variable morbidity in ferrets, but also showed no correlation between the ability to infect the ferrets and the source of the virus (e.g. wild bird vs. poultry) [7]. This study provides additional indication that the source of virus may not be as important of a factor in transmission to mammals, regardless of subtype, although many other factors would play a role in natural transmission including host interactions and amount of virus shed.

Avian influenza virus infections and H7 infectious in particular have presented as conjunctivitis [2]. While this route of administration is understudied in ferrets, Belser et al [48] recently explored infection of ferrets with human influenza viruses and avian influenza viruses isolated from human infections. Both human and avian-origin influenza viruses, including subtypes H1, H3, H5, and H7, and HPAI and LPAI types could infect via the ocular route and spread to the pulmonary tract. Interestingly, the H7 influenzas transmitted to naïve contact ferrets [48]. We did not explore ocular infection in this study; however in light of the demonstrated infection and transmission with H7 influenza viruses, this should be an area of future study.

Pulmonary replication of these two AIVs was rapid in onset and rapid to resolve regardless of magnitude of virus and pulmonary lesions. This rapid transient infection may be related to inoculation methods, which place a large dose of virus in an anesthetized ferret where it becomes inhaled deep into the respiratory tract. This confounds risk assessment, as natural exposure would be through a fomite or aerosol droplet from another infected individual. Early pulmonary viral titers and histopathology have not been examined in transmission ferrets in other transmission experiments or in this study. This would be an interesting component to evaluate to aid in determining the pulmonary replication capacity of these AIVs in a more realistic transmission setting.

Few studies have examined hematologic parameters in influenza infected ferrets, but investigation into this is worthy as it may be a good measure of clinical disease [49]. Examining clinical pathologic findings of such a small sample size can be difficult, given the marked variation between individuals. Nonetheless, there was a trend in both groups H6N1 and H1N9 compared to the allantoic inoculated group of a decrease in lymphocytes on day 1 pi that resolved by day 3 pi for individual animals. Lymphopenia is well established to occur in the very early stages of viral infections. Indeed, it has been demonstrated that experimental ferret infections with HPAI H5N1 viruses result in a profound lymphopenia days 3 and 5 pi [50]. Also, ferrets infected with a variety of H1N1 influenza viruses had a decrease in lymphocytes days 3 and 7 pi [49]. For H1N9 inoculated animals, lymphocytes were increased between days 7 pi through 18 pi for many individuals. Lymphocytosis has not been described in ferrets experimentally infected with influenza, although this finding seems logical, given that chronic antigenic stimulation can induce increases in lymphocytes resulting in peripheral lymphocytosis.

Infectivity of AIVs in mammals and humans is thought to be reliant on the viral hemagglutinin binding sialic acid (SA) residues on host cells, and differences in binding between mammalian versus avian influenza viruses are suggested to be partially responsible for host specificity and localization of infection [24], [51]. It is thought that the avian influenza preference for binding α2,3 linked SA receptors compared to the human influenza preference for binding α2,6 linked SA receptors provides somewhat of a barrier to transmission in ferrets and humans due to the paucity of α2,3 linked SA in the upper respiratory tract. However, there is a presence of α2,3 linked SA in the lower respiratory tract of both these species, and it has been proposed that if enough influenza can be deposited in the lower respiratory tract, pulmonary infection will predominate in these species [47]. We did observe infection and replication in the lung of these ferrets for both H6N1 and H1N9 that supports viral attachment in the lung (bronchioles, but not alveoli), however, we also observed robust upper respiratory tract infections that were more productive with higher viral titers present than compared to the lung despite the alpha α2,3 SA binding preference (Table 1, Figures 1–2).We and others typically observe that ferrets infected with human influenza viruses have viral replication restricted to the upper respiratory tract [52]–[54]. The absence of pulmonary infection is confirmed by negative virus isolation, absence of lesions on histopathology, and absence of viral antigen on IHC. Very mild pulmonary infections have been demonstrated in other laboratories with human influenza viruses in experimental ferret infections, however severe pneumonia is only associated with highly virulent human influenza viruses or highly pathogenic avian influenza viruses [38], [47], [55]. In this study, neither H6N1 nor H1N9 virus appeared to infect alveolar epithelial cells (Figure 2) yet, both viruses did infect mouse and feline alveolar epithelial cells in other *in vivo* experimental trials in our laboratory (submitted and [19]). Other experiments have demonstrated replication of AIVs in the upper and lower respiratory tract of ferrets with some viruses having higher replication in the nasal turbinates and others with higher replication in the lung [6], [7], [21]. Clearly, cellular tropism in an influenza infected host is complex, and while the cellular SA ligand for HA binding is certainly an important component, additional mechanisms are likely at work.

In our study, the H1N9 subtype AIV exhibited efficient direct contact transmission between ferrets. Aerosol transmission was not explored in this study due to lack of a validated aerosol transmission model in our facilities, however, it would be an interesting next step. Many experiments have established the importance of receptor binding in influenza transmission, demonstrating limited or no contact or aerosol transmission in viruses that have a binding preference for α2,3 linked SA over α2,6 linked SA [10], [43], [56], [57]. Both the H1N9 and H6N1 viruses have avian specific α 2,3 linked SA receptor binding as shown in the erythrocyte binding assays. There are no previously defined HA amino acid residues that would suggest an altered receptor specificity and this assumption is supported by the glycan microarray analysis, where H1N9 dominantly bound glycans having α2,3 SA linkages. However, there may be other unidentified amino acids in the HA or other viral gene segments that mediated the efficient direct transmission of the H1N9 in ferrets, where other H1N1 viruses failed to efficiently transmit via contact [43]. Direct contact H6N1 ferrets did not become infected and did not seroconvert (Figure 1). Perhaps this is due to lower levels of viral shedding for a shorter period of time in H6N1 infected ferrets as compared to H1N9 infected ferrets.

The viral polymerase has also been suggested to potentially have a role in efficient avian to mammalian transmission, replication, and localization of viral infection based upon differences in temperature for optimal replication, tissue/species tropism for replication, rate of replication, and effect on efficiency of viral nuclear transport [43], [58]–[64]. Sequence analysis of H1N9 PB2 found avian specific Glu627 and Asp701 residues (GenBank Accession ACY79819; data not shown), suggesting that there may be other genetic features contributing to the robust upper respiratory replication and transmission of this H1N9 virus in ferrets.

Our study, in combination with additional studies of AIV infections in ferrets, indicates that there is a capacity for wild bird AIVs, subtype notwithstanding, to directly infect mammals with minimal clinical signs. The results support the potential for direct interspecies transmission or formation of a viable AIV reassortant. Although we have demonstrated the low virulence and rapid clearance of these AIVs, possibilities for reassortment in susceptible wild and domestic mammalian species make these species of particular interest and worth further investigation. Furthermore, the variable magnitude of seroconversion despite productive influenza infection could make surveillance and monitoring for mammalian infection with AIVs difficult. Together, these studies support the need for expanded analysis of influenza viruses from their reservoir species as understanding of the mechanisms of infection and transmission is incomplete and subsequent risk assessment imperfect.

## Materials and Methods

### Ethics statement

These studies were conducted in strict accordance with guidelines approved by the Institutional Animal Care and Use Committee and supported through the Office of Animal Care and use of the University of Georgia, following guidelines established by AAALAC International (Association for Assessment and Accreditation of Laboratory Animal Care; Accreditation Date: 3/2/2011), licensed by the USDA (USDA #57-R-005), and maintaining an Assurance of Compliance with the U.S. Department of Health and Human Services (PHS Assurance #A3437-01). The protocols utilized for these studies were approved by the Institutional Animal Care and Use Committees of the University of Georgia and the Centers for Disease Control and Prevention.

### Viruses

Avian influenza viruses used were cloacal swab isolates from wild birds in the United States acquired from Southeastern Cooperative Wildlife Disease Study (Athens, GA). Viruses were isolated from cloacal swabs in 9 day old embryonated chicken eggs (ECE) at 37°C for 72 hours and then minimally passaged (3 or fewer passages) in ECEs. Select virus isolates were screened in a previous study in BALB/c mice [19]. Two viruses that exhibited efficient pulmonary replication and induced pulmonary lesions in BALB/c mice were selected for *in vivo* studies in ferrets: A/Ruddy Turnstone/DE/892/02 (H6N1 subtype; NCBI Taxonomy ID: 680602; abbreviated H6N1) and A/Ruddy Turnstone/DE/1171/02 (H1N9 subtype; NCBI Taxonomy ID: 680596; abbreviated H1N9). The original low passage isolates, once selected by screening methods, were grown once more in 9 to 10 day old ECE to generate a stock of the virus. Stock viruses were aliquoted and stored at −80°C until use. Stock virus titers were determined by plaque assay on MDCK cells.

### Sequencing

Total viral RNA was extracted from AIV infected allantoic fluid using the RNeasy kit (QIAGEN, Inc., Valencia, CA) according to the manufacturer's protocol. One-step RT-PCR was performed on viral RNA using a universal primer set (Uni12/Inf-1 5′-GGGGGGAGCAAAAGCAGG-3′ and Uni13/Inf-1 5′-CGGGTTATTAGTAGAAACAAGG-3′) as previously described [65]. All 8 segments were generated, the HA segment was excised and gel purified using the QIAquick Gel Extraction kit (QIAGEN, Inc., Valencia, CA). The HA was sequenced using BigDye Terminator v3.1 Cycle Sequencing kit (Applied Biosystems) with subtype specific primers (primer sequences available upon request). Some sequences for the H1N9 and H6N1 viruses were already available on GenBank. The accession numbers are GU050646.1, GU050647.1, GU050624.1, GU050623.1, GU050622.1, GU050620.1, GU050619.1, GU050618.1, and GU050621.1 (A/ruddy turnstone/Delaware/892/2002 segment 1 and segment 2, and A/ruddy turnstone/Delaware/1171/2002 segments, 1, 2, 3, 5, 6, 7, and 8 respectively). HA sequences are available at GenBank. Segments sequenced for this study and submitted to GenBank include A/ruddy turnstone/Delaware/892/2002 segments 3, 4, 6, and 8 and A/ruddy turnstone/Delaware/1171/2002 segment 4 (GenBank Accession numbers CY116632, CY116633, CY116634, CY116633, and CY116631, respectively).

### Erythrocyte binding assays

Fresh turkey, guinea pig, and equine erythrocytes were thoroughly washed with 1× PBS and resuspended to 1% v/v in 1× PBS/.5%BSA. A standard hemagglutination assay was performed for each AIV isolate against all types of erythrocytes, and appearance of agglutination was scored after a 60-min incubation period.

### Glycan microarray binding

Viruses were cultured at an MOI of 0.01 on MDCKs for 72 hours in 1× Minimal Essential Medium supplemented with 1 µg/ml TPCK [L-(tosylamido-2-pheyl) ethyl chloromethyl ketone]-treated trypsin (Worthington Biochemical Corporation, Lakewood, NJ). The viral supernatant (10 ml) was collected and centrifuged at 5,000 RPM for 5 minutes to remove cell debris before viral purification. Virus in the culture supernatant was purified on a 25% sucrose cushion and resuspended in 1× PBS with 1 mM EDTA. Briefly, each virus was purified through a 25% sucrose gradient by high speed centrifugation at 28,000 rpm at 4°C for 3 hours. The purified viruses were resuspended in1XPBS with 1 mM EDTA on ice for 4 hours and stored at −80°C. Viral titers were determined by standard plaque assay on MDCK cells. Approximately 10^7^ PFU of each purified strain were labeled with 25 µg of Alexa488 dye in 1 M NaHCO_3_ (pH 9) for 1 hour. To remove residual dye, each sample was dialyzed in a 7000 MWCO Slide-A-Lyzer MINI dialysis cassette (Thermo Scientific) against PBS with 1 mM EDTA overnight. The labeled viruses were analyzed via glycan microarray by the Core H of the Consortium of Functional Glycomics (www.functionalglycomics.org), where 70 µl of labeled virus was added to glycan microarray slide and incubated at 4°C for 1 hour. Each microarray was scanned by Perkin-Elmer ProScanAray that detected SA binding peaks designated as relative fluorescent units (RFUs).

### Ferrets

Castrated male Fitch ferrets (Triple F Farms, Sayre, PA), 3 months old and seronegative to circulating human H1N1 and H3N2 influenza viruses, were used for the study. Ferrets were housed in a BSL2 facility in HEPA filtered isolator caging (Allentown, Allentown, NJ). A subcutaneous temperature transponder (BMDS, Seaford, DE) was implanted in each ferret for identification and temperature measurement.

### Infection and direct transmission study

Seven ferrets were inoculated per virus (four for tissue examination and three for transmission study) and three additional naive ferrets were used to assay direct contact transmission. In the contact trials, one direct contact ferret was housed with one inoculated ferret as paired cage mates. An additional three ferrets were mock infected with allantoic fluid in PBS for negative controls for nasal washes, serology, and complete blood counts (CBC). Inoculated ferrets were lightly anesthetized with isoflurane and intranasally inoculated with 5×10^5^ PFU in 500 µL of sterile PBS (250 µL of per nostril) with either H6N1 or H1N9. Direct contact ferrets were placed with inoculated ferrets twenty-four hours post inoculation. An additional study was performed with H1N9 as previously described to confirm direct contact transmission, using three inoculated and three direct contact ferrets. Temperatures monitored for four days to establish baseline then temperature, weights, and complete blood counts were monitored in inoculated ferrets on days 1, 3, 5, 7, 10, 13, 18, and 21 pi and in direct contact ferrets on days 2, 4, 6, 9, 12, 17, and 20 post contact (pc). Nasal washes were sampled from ferrets on days 1, 3, 5, 7, 10 pi or days 2, 4, 6, 9, 12, and 17 pc to monitor for viral infection. For nasal washes, ferrets were anesthetized with 4 mg ketamine via intramuscular injection and 1 mL of sterile PBS with penicillin (4000 U/ml) (Calbiochem, Gibbstown, NJ), streptomycin (800 µg/ml) (Sigma, St. Louis, MO), polymyxin B (400 U/ml) (MP Biochmemicals, LLC, Solon, OH), and gentamicin (100 µg/ml) (Gibco, Carlsbad, CA) was introduced into the nostrils to induce sneezing and collected in specimen cups. For repeat study ferrets, temperature, weights, and nasal washes were performed for inoculated ferrets and transmission ferrets on days 1, 3, 5, 7, 9, 11, 13 and 15 pi and on days 2, 4, 6, 8, 10, 12, and 14 pc. Repeat study ferrets were humanely euthanized at day 21 pi (day 20 pc) and samples of lung from all ferrets were fixed in neutral buffered formalin for histopathology.

### Determination of viral titers

Nasal washes were immediately tested via real time RT-PCR to aid in determining days for sample collection. Briefly, viral RNA was extracted from nasal wash by using RNeasy mini kit (QIAGEN, Inc., Valencia, CA) and the Qiagen one-step RT-PCR kit was used for RRT-PCR with a Stratagene MX300P/3005P thermocyler and Mx Pro QPCR software (La Jolla, CA). Reaction mixture and PCR cycling protocol is available upon request. An influenza virus matrix gene specific primer and probe set were used as follows: primer M+25, sequence AGA TGA GTC TTC TAA CCG AGG TCG; primer M-124, sequence TGC AAA AAC ATC TTC AAG TCT CTG; and probe M+64, sequence FAM-TCA GGC CCC CTC AAA GCC GA-TAMRA [66] (Biosearch Technologies, Novato, CA).

Four ferrets per virus were humanely euthanized (two on day 3 pi and two on day 7 pi per virus) and lung, nasal turbinate, liver, spleen, and olfactory bulb were sampled under sterile conditions and frozen at −80°C for virus isolation. Based on histopathology and viral titers from the lung, we suspected early pulmonary infection with rapid clearance, therefore an additional two ferrets were intranasally inoculated as previously described with H1N9 and humanely euthanized at day 2 pi with fresh lung collected to examine the earlier time point. Tissues were later homogenized in 1 mL PBS with antibiotics, clarified by centrifugation, and 100 µL of clarified homogenate was inoculated into 9 to 10 day old ECEs for virus isolation (4 eggs per sample, 72 hour incubation). Nasal washes and clarified lung homogenate were titrated in MDCK cells with serial 1∶10 or 1∶3 dilutions with a 1.5 log_10_TCID_50_/mL (nasal wash) and 1. 3 to 1.0 log_10_ TCID_50_/gram (lung).

### Serology and hematology

At day 21 pi (day 20 pc), blood was collected and seroconversion was determined via hemagluttination inhibition (HI) and microneutralization (MN) assays. All blood samples were analyzed the same day as sample collection. Complete blood counts were performed using VetScan analyzer (Abaxis, Union City, CA). Leukocyte counts were log transformed and statistically analyzed using repeated measures ANOVA (Stata version 11.0) to examine differences between viral groups and days pi. Degrees of freedom for F-tests of repeated measures factors were adjusted using the Greenhouse-Geisser estimate of epsilon to correct for any departures from the sphericity assumption. All testing assumed a two-sided alternative hypothesis and P-values<0.05 were considered significant.

### Histopathology and immunohistochemistry

Lung (cranial and caudal lobes), trachea, tracheobronchial lymph node, esophagus, heart, spleen, liver, stomach, small intestine, large intestine, pancreas, mesenteric lymph node, kidneys, adrenal gland, bladder, brain, and nasal turbinates were collected on days 3 and 7 pi from inoculated ferrets (two ferrets per virus per day, the same ferrets as described for virus isolation in fresh tissues). The additional two ferrets that were intranasally inoculated with H1N9 and humanely euthanized at day 2 pi had the same set of tissues collected for histopathology and immunohistochemistry. All tissues were preserved in 10% neutral buffered formalin. Tissues were routinely processed, embedded and stained with hematoxylin and eosin. Immunohistochemical staining was performed on lung, trachea, and nasal turbinates for all ferrets. Immunohistochemistry was performed using a commercially available goat polyclonal antibody to the nucleoprotein of influenza A virus at a 1∶10,000 dilution (Biodesign International, Sako, Maine). Tissues were deparaffinized and blocked with a commercial protein blocking agent (Dako Cytomation, Carpinteria, CA) and a linked strepavidin-biotin immunoperoxidase system was used for immunolabeling. The reaction was visualized with 3, 3′-diaminobenzidine substrate (Dako Cytomation, Carpinteria, CA).

## Supporting Information

Table S1 **Total and differential leukocyte counts for ferrets inoculated with wild bird avian influenza viruses H1N9 and H6N1.**
(DOCX)

Table S2 **Raw Glycan Microarray Data.**
(DOCX)
