# Supplementary material for: Low Pathogenic Avian Influenza Isolates from Wild Birds Replicate and Transmit via Contact in Ferrets without Prior Adaptation
Source: PLoS One. 2012 Jun 1;7(6):e38067. doi: 10.1371/journal.pone.0038067 (PMC3365887; doi:10.1371/journal.pone.0038067)
Supplement: Table S1 — Total and differential leukocyte counts for ferrets inoculated with wild bird avian influenza viruses H1N9 and H6N1. (DOCX) [file pone.0038067.s001.docx]

Table S1. Total and differential leukocyte counts for ferrets inoculated with wild bird avian influenza viruses H1N9 and H6N1.

|  |  | Individual ferret leukocyte counts, 3 ferret per group | | | | | | | | | | |
| --- | --- | --- | --- | --- | --- | --- | --- | --- | --- | --- | --- | --- |
|  | Day pi | Allantoic Fluid | | |  | H6N1 | | |  | H1N9 | | |
| Total Leukocytes (cells/uL) |  |  |  |  |  |  |  |  |  |  |  |  |
|  | -2 | 6410 | 8760 | 8300 |  | 7170 | 7710 | 8880 |  | 10300 | 18760 | 12290 |
|  | 1 | 8480 | 8910 | 5870 |  | 6310 | 7740 | 5040 |  | 7060 | 11110 | 11200 |
|  | 3 | 8960 | 10070 | 11080 |  | - | 6030 | 11300 |  | 13020 | 17430 | 13280 |
|  | 5 | 12970 | 7880 | 10860 |  | 8410 | 10780 | 6500 |  | 12840 | 13740 | 18430 |
|  | 7 | 10840 | 10730 | 10230 |  | 9020 | 7960 | 9530 |  | 35460 | 19490 | 13430 |
|  | 10 | 8900 | 10300 | 12170 |  | 6560 | 7630 | 8270 |  | 15920 | 20730 | 13960 |
|  | 13 | 13970 | 6850 | 11770 |  | 12020 | 10640 | 6890 |  | 10000 | 18090 | 20550 |
|  | 18 | 22160 | 12960 | 13640 |  | 21170 | 10470 | 16950 |  | 25250 | 19650 | 12520 |
|  | 21 | 13450 | 11420 | 6070 |  | 8390 | 8050 | 12660 |  | 9620 | 5950 | 13420 |
| Lymphocytes (cells/uL) |  |  |  |  |  |  |  |  |  |  |  |  |
|  | -2 | 2340 | 3570 | 8300 |  | 2850 | 2620 | 4210 |  | 4420 | 7180 | 6090 |
|  | 1 | 2890 | 3560 | 2140 |  | 1670 | 2360 | 1550 |  | 2760 | 5720 | 4280 |
|  | 3 | 3730 | 3860 | 2610 |  | - | 3200 | 4650 |  | 7130 | 8210 | 7500 |
|  | 5 | 5410 | 3270 | 2470 |  | 3760 | 420 | 2990 |  | 6240 | 5860 | 8620 |
|  | 7 | 6190 | 5890 | 2780 |  | 4030 | 3770 | 5140 |  | 24130 | 8660 | 7240 |
|  | 10 | 3600 | 4300 | 3720 |  | 2380 | 3090 | 4280 |  | 7450 | 12480 | 6680 |
|  | 13 | 7540 | 3640 | 4900 |  | 6890 | 5930 | 4020 |  | 6430 | 9370 | 11500 |
|  | 18 | 12200 | 6090 | 4760 |  | 11550 | 5150 | 10190 |  | 14930 | 11250 | 6330 |
|  | 21 | 6290 | 5950 | 2480 |  | 3620 | 4210 | 9330 |  | 5340 | 4160 | 7350 |
| Granulocytes (cells/uL) |  |  |  |  |  |  |  |  |  |  |  |  |
|  | -2 | 3790 | 5120 | 5340 |  | 3390 | 5030 | 4560 |  | 5260 | 10050 | 6120 |
|  | 1 | 4710 | 5280 | 3630 |  | 4060 | 4520 | 3100 |  | 3740 | 3870 | 5930 |
|  | 3 | 4050 | 6140 | 7980 |  | - | 4530 | 6570 |  | 4540 | 6870 | 5680 |
|  | 5 | 7470 | 4540 | 7460 |  | 3640 | 5130 | 2890 |  | 5130 | 6020 | 9680 |
|  | 7 | 3800 | 4750 | 7370 |  | 4920 | 4140 | 4330 |  | 11120 | 8260 | 5650 |
|  | 10 | 5240 | 5280 | 8370 |  | 3660 | 3780 | 2970 |  | 8360 | 6560 | 7180 |
|  | 13 | 5890 | 3170 | 5930 |  | 3530 | 4640 | 2820 |  | 4140 | 6800 | 7290 |
|  | 18 | 8250 | 6560 | 8120 |  | 9470 | 5180 | 5890 |  | 10170 | 7920 | 5270 |
|  | 21 | 7070 | 5400 | 2930 |  | 3650 | 3680 | 3250 |  | 3400 | 1610 | 5990 |
