# Supplementary material for: Low Pathogenic Avian Influenza Isolates from Wild Birds Replicate and Transmit via Contact in Ferrets without Prior Adaptation
Source: PLoS One. 2012 Jun 1;7(6):e38067. doi: 10.1371/journal.pone.0038067 (PMC3365887; doi:10.1371/journal.pone.0038067)
Supplement: Table S2 — Raw Glycan Microarray Data. (DOCX) [file pone.0038067.s002.docx]

Table S2:  Raw Glycan Microarray Data


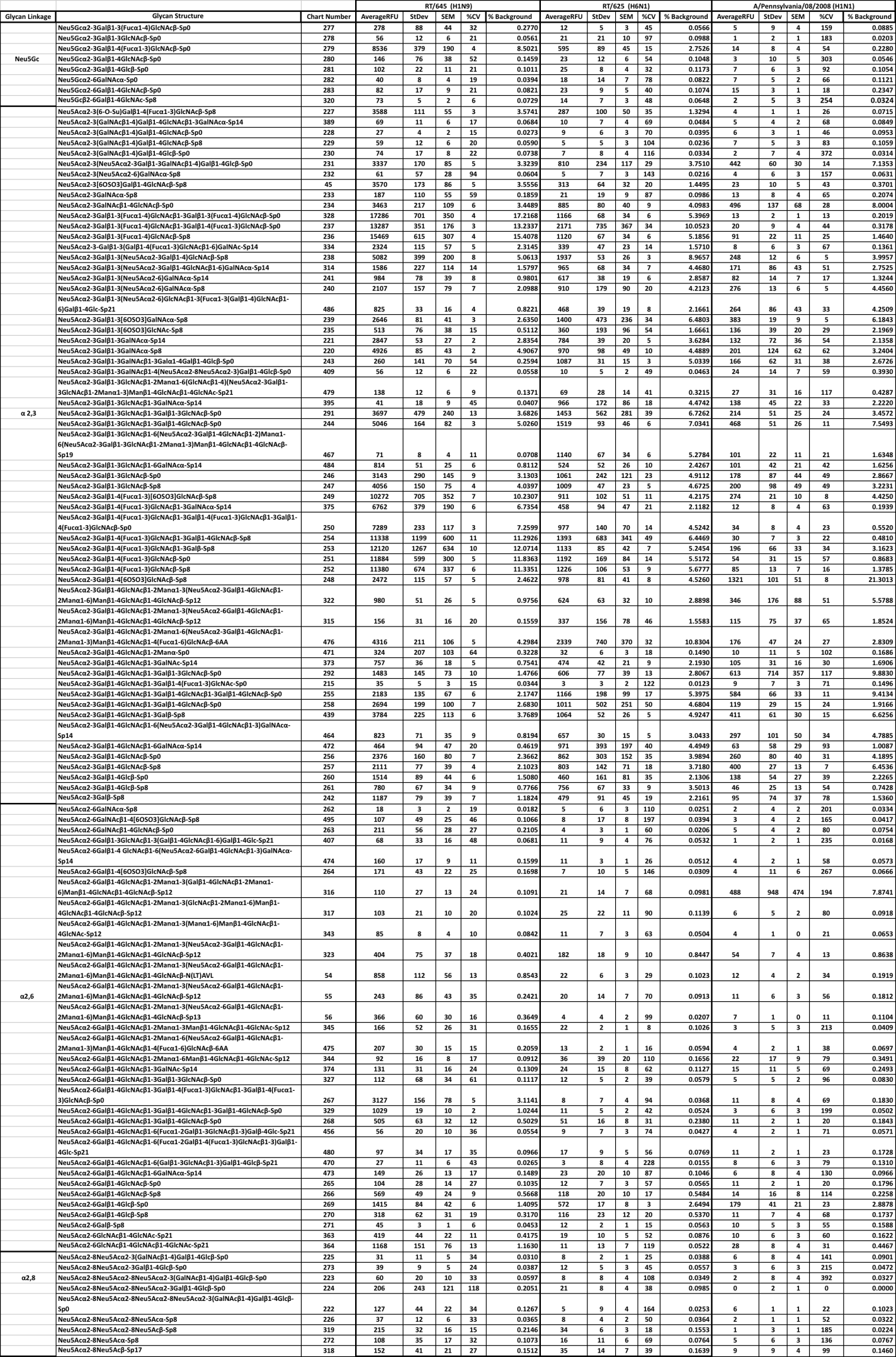


The N‐glycolylneuraminic acid ( Neu5GC) and N‐acetyl‐neuraminic acid (Neu5Ac) glycans were included on the Version 4.2 microarray, where the α2,3 linked, α2,6 linked, and α2,8 linked SA were illustrated in Figure 3. The average relative fluorescence units (RFU), standard deviation (STDev), standard error of the mean (SEM), % coefficient of variation (%CV), and % background were generated for the RT/645, RT/625, and A/Pennsylvania/08/2008 strains, where the % background was determined by the average RFU divided by the average RFU for 511 glycans multiplied by two.
